# Supplementary material for: Community Structure and Diversity of Endophytic Fungi in Cultivated Polygala crotalarioides at Two Different Growth Stages Based on Culture-Independent and Culture-Based Methods
Source: J Fungi (Basel). 2024 Mar 4;10(3):195. doi: 10.3390/jof10030195 (PMC10970964; doi:10.3390/jof10030195)
Supplement: Supplementary file 1 [file jof-10-00195-s001.zip › Figure S1.pdf]

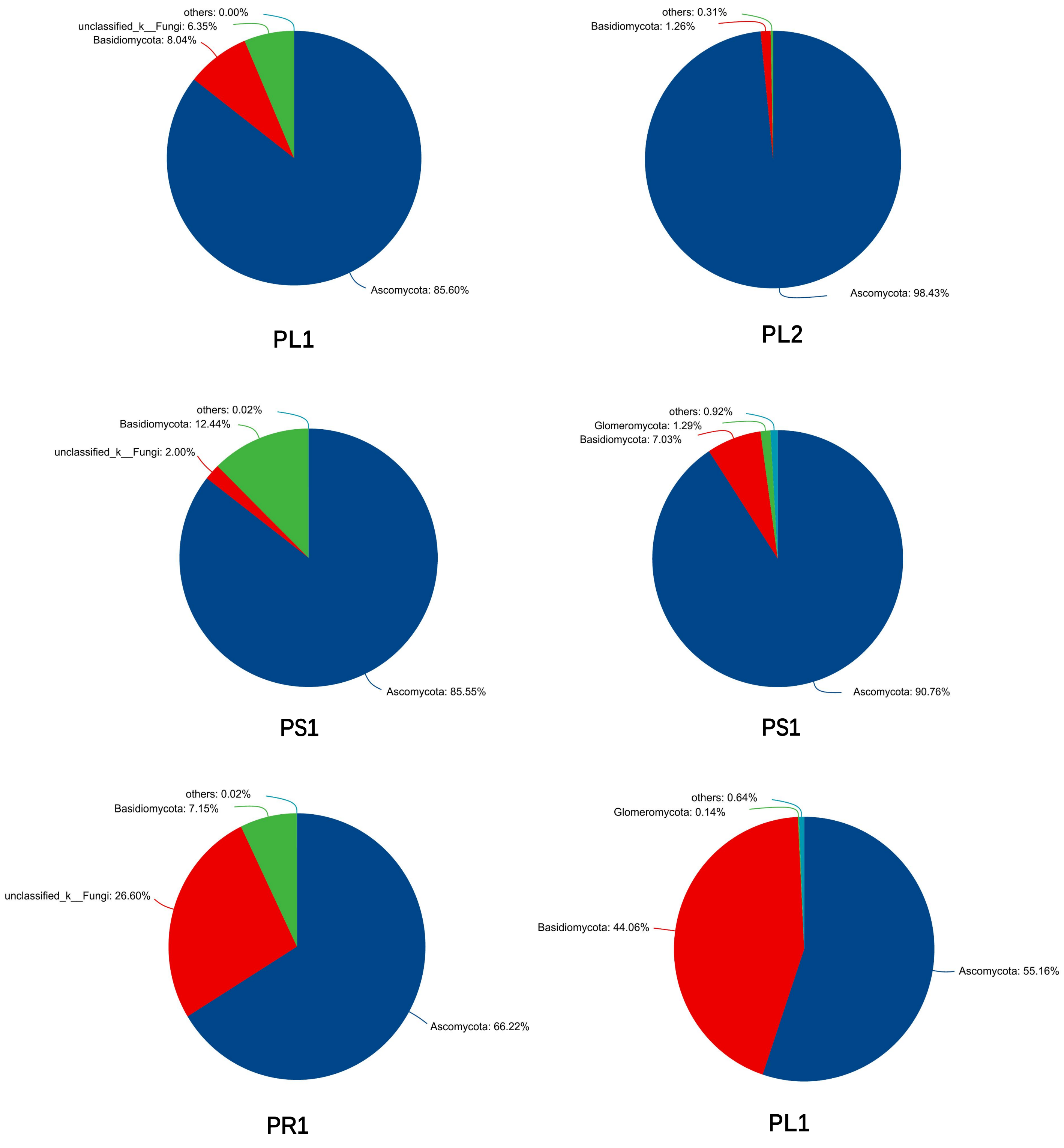

Figure S1. Community analysis pielot of endophytic fungi from *P. crotalarioides* at Phylum level.
