# Supplementary material for: Community Structure and Diversity of Endophytic Fungi in Cultivated Polygala crotalarioides at Two Different Growth Stages Based on Culture-Independent and Culture-Based Methods
Source: J Fungi (Basel). 2024 Mar 4;10(3):195. doi: 10.3390/jof10030195 (PMC10970964; doi:10.3390/jof10030195)
Supplement: Supplementary file 1 [file jof-10-00195-s001.zip › Table S4.pdf]

Table S4. Comparison of community structure of endophytic fungi detected with high-throughput sequencing at each classification level.

sample	source	Phylum	Class	Order	Family	Genus	Species	OTU
1-year sample	leaf	4	15	34	68	99	121	204
	stem	4	16	36	75	99	113	164
	root	5	17	30	48	55	57	121
	total	7	21	49	103	156	191	355
2-year sample	leaf	3	13	30	61	92	128	218
	stem	5	24	68	161	276	418	829
	root	7	18	33	59	86	102	143
	total	7	27	74	175	323	489	981
