# Supplementary material for: Community Structure and Diversity of Endophytic Fungi in Cultivated Polygala crotalarioides at Two Different Growth Stages Based on Culture-Independent and Culture-Based Methods
Source: J Fungi (Basel). 2024 Mar 4;10(3):195. doi: 10.3390/jof10030195 (PMC10970964; doi:10.3390/jof10030195)
Supplement: Supplementary file 1 [file jof-10-00195-s001.zip › Table S5.pdf]

**Table S5.** Taxonomic information of all endophytic fungi isolated from *P. crotalarioides*.

| source | strain No. | Species | phylum | class | order | family |
|---|---|---|---|---|---|---|
| 1-year leaf | PCAM095 | *Coniochaeta velutina* | Ascomycota | Sordariomycetes | Hypocreales | Coniochaetaceae |
| | PCAM033 | *Didymella bellidis* | Ascomycota | Dothideomycetes | Pleosporales | Didymellaceae |
| | PCAM078 | *Didymella bellidis* | Ascomycota | Dothideomycetes | Pleosporales | Didymellaceae |
| | PCAM091 | *Didymella bellidis* | Ascomycota | Dothideomycetes | Pleosporales | Didymellaceae |
| | PCAM003 | *Fusarium tricinctum* | Ascomycota | Sordariomycetes | Hypocreales | Nectriaceae |
| | PCAM074 | *Fusarium tricinctum* | Ascomycota | Sordariomycetes | Hypocreales | Nectriaceae |
| | PCAM073 | *Pezicula chiangraiensis* | Ascomycota | Leotiomycetes | Helotiales | Dermateaceae |
| | PCAM010 | *Phialophora mustea* | Ascomycota | Eurotiomycetes | Chaetothyriales | Herpotrichiellaceae |
| | PCAM034 | *Phialophora* sp. | Ascomycota | Eurotiomycetes | Chaetothyriales | Herpotrichiellaceae |
| | PCAM087 | *Talaromyces funiculosus* | Ascomycota | Eurotiomycetes | Chaetothyriales | Trichocomaceae |
| 1-year stem | PCAP041 | *Alternaria alternata* | Ascomycota | Dothideomycetes | Pleosporales | Pleosporaceae |
| | PCAR020 | *Coniochaeta velutina* | Ascomycota | Sordariomycetes | Hypocreales | Coniochaetaceae |
| | PCAP033 | *Fusarium petersiae* | Ascomycota | Sordariomycetes | Hypocreales | Nectriaceae |
| | PCAP003 | *Fusarium reticulatum* | Ascomycota | Sordariomycetes | Hypocreales | Nectriaceae |
| | PCAP096 | *Fusarium reticulatum* | Ascomycota | Sordariomycetes | Hypocreales | Nectriaceae |
| | PCAP012 | *Geotrichum candidum* | Ascomycota | Saccharomycetes | Saccharomycetales | Dipodascaceae |
| | PCAP036 | *Juxtiphoma* sp. | Ascomycota | Dothideomycetes | Pleosporales | Didymellaceae |
| | PCAP093 | *Pezicula sporulosa* | Ascomycota | Leotiomycetes | Helotiales | Dermateaceae |
| | PCAP001 | *Phialophora mustea* | Ascomycota | Eurotiomycetes | Chaetothyriales | Herpotrichiellaceae |

| | | | | | | |
|---|---|---|---|---|---|---|
| 1-year root | PCBM001 | *Botrytis cinerea* | Ascomycota | Leotiomycetes | Helotiales | Sclerotiniaceae |
| | PCBM035 | *Botrytis cinerea* | Ascomycota | Leotiomycetes | Helotiales | Sclerotiniaceae |
| | PCBR020 | *Cladosporium* sp. | Ascomycota | Dothideomycetes | Pleosporales | Cladosporiaceae |
| | PCBR013 | *Colletotrichum acutatum* | Ascomycota | Sordariomycetes | Hypocreales | Glomerellaceae |
| | PCBM022 | *Colletotrichum dematium* | Ascomycota | Sordariomycetes | Hypocreales | Glomerellaceae |
| | PCBM027 | *Diaporthe nobilis* | Ascomycota | Sordariomycetes | Hypocreales | Diaporthaceae |
| | PCBP027 | *Talaromyces muroii* | Ascomycota | Eurotiomycetes | Chaetothyriales | Trichocomaceae |
| 2-year leaf | LM17 | *Alternaria alternata* | Ascomycota | Dothideomycetes | Pleosporales | Pleosporaceae |
| | LM80 | *Alternaria blumeae* | Ascomycota | Dothideomycetes | Pleosporales | Pleosporaceae |
| | LR87 | *Colletotrichum boninense* | Ascomycota | Sordariomycetes | Glomerellales | Glomerellaceae |
| | LR51 | *Colletotrichum gloeosporioides* | Ascomycota | Sordariomycetes | Glomerellales | Glomerellaceae |
| | LP24 | *Colletotrichum horii* | Ascomycota | Sordariomycetes | Glomerellales | Glomerellaceae |
| | LP41 | *Fusarium oxysporum* | Ascomycota | Sordariomycetes | Hypocreales | Nectriaceae |
| | LM35 | *Leptosphaerulina arachidicola* | Ascomycota | Dothideomycetes | Pleosporales | Didymellaceae |
| | LP34 | *Nigrospora chinensis* | Ascomycota | Sordariomycetes | Xylariales | Apiosporaceae |
| | LR43 | *Nigrospora chinensis* | Ascomycota | Sordariomycetes | Xylariales | Apiosporaceae |
| | LR62 | *Nigrospora osmanthi* | Ascomycota | Sordariomycetes | Xylariales | Apiosporaceae |
| | LR101 | *Nigrospora rubi* | Ascomycota | Sordariomycetes | Xylariales | Apiosporaceae |
| | LP01 | *Penicillium brefeldianum* | Ascomycota | Eurotiomycetes | Eurotiales | Aspergillaceae |
| | LP851 | *Penicillium brefeldianum* | Ascomycota | Eurotiomycetes | Eurotiales | Aspergillaceae |
| | LP87 | *Pyronema omphalodes* | Ascomycota | Pezizomycetes | Pezizales | Pyronemataceae |

| | | | | | | |
|---|---|---|---|---|---|---|
| 2-year stem | SR58 | *Chaetomium cochliodes* | Ascomycota | Sordariomycetes | Sordariales | Chaetomiaceae |
| | SR60 | *Fusarium oxysporum* | Ascomycota | Sordariomycetes | Hypocreales | Nectriaceae |
| | SM21 | *Nigrospora chinensis* | Ascomycota | Sordariomycetes | Xylariales | Apiosporaceae |
| | SM40 | *Nigrospora rubi* | Ascomycota | Sordariomycetes | Xylariales | Apiosporaceae |
| | SM79 | *Pestalotiopsis kenyana* | Ascomycota | Sordariomycetes | Xylariales | Sporocadaceae |
| | SM81 | *Phoma herbarum* | Ascomycota | Dothideomycetes | Pleosporales | Didymellaceae |
| | SM04 | *Schizophyllum commune* | Basidiomycota | Agaricomycetes | Agaricales | Schizophyllaceae |
| 2-year root | FR89 | *Alternaria alternata* | Ascomycota | Dothideomycetes | Pleosporales | Pleosporaceae |
| | RR09 | *Aspergillus* sp. | Ascomycota | Eurotiomycetes | Eurotiales | Aspergillaceae |
| | FM25 | *Colletotrichum horii* | Ascomycota | Sordariomycetes | Glomerellales | Glomerellaceae |
| | FP76 | *Diaporthe unshiuensis* | Ascomycota | Sordariomycetes | Diaporthales | Diaporthaceae |
| | RM07 | *Epicoccum nigrum* | Ascomycota | Dothideomycetes | Pleosporales | Didymellaceae |
| | FM83 | *Epicoccum sorghinum* | Ascomycota | Dothideomycetes | Pleosporales | Didymellaceae |
| | FR92 | *Periconia pseudobyssoides* | Ascomycota | Dothideomycetes | Pleosporales | Periconiaceae |
| | RM06 | *Schizophyllum commune* | Basidiomycota | Agaricomycetes | Agaricales | Schizophyllaceae |
